# Effects of arm weight and target height on hand selection: A low-cost virtual reality paradigm

**DOI:** 10.1371/journal.pone.0207326

**Published:** 2019-06-21

**Authors:** Eric James McDermott, Marc Himmelbach

**Affiliations:** 1 Center of Neurology, Division of Neuropsychology, Hertie-Institute for Clinical Brain Research, University of Tübingen, Tübingen, Germany; 2 Center of Neurology, Department Neurology and Stroke, Hertie-Institute for Clinical Brain Research, University of Tübingen, Tübingen, Germany; University of Exeter, UNITED KINGDOM

## Abstract

We evaluated the ability of a virtual reality (VR) system to reliably detect the reaching frequency midline position of a user; the distinguishing plane between free-choice use of the left and right hand. The paradigm utilized the Leap Motion Hand Tracker along with a custom script written in C# and was realized through a Unity3D application. Stimuli appeared in random locations on the computer screen and required the participant to reach with the hand of their choice to contact them with a virtually coupled hand inside the virtual space. We investigated the effects of two manipulations of effort on the free-choice reaching of either the left or right hand. We varied the height of target positions and applied an additional weight to the non-dominant, left hand. We observed main effects of height and weight on reaching frequency midline positions across the group. We found increased use of the dominant hand as stimuli height increased, as well as a significant increase in overall use of the dominant, right hand when a weighted-glove was worn by the non-dominant, left hand. Our results are in line with previously published research on hand selection from similar paradigms, supporting the use of our VR paradigm in future experiments and applications.

## Introduction

Handedness is one of the most obvious asymmetries in the world’s population, with around ~90% identifying as “right-handers” [1]. An individual’s selection of the dominant or non-dominant hand is not only driven by a constant limb preference but is context and task dependent [2–6].

Gabbard et al. [7] found a strong (>95%) preference for the dominant hand at body midline target positions and in ipsilateral space. This dominant hand preference dropped to under 50% once the targets crossed into contralateral space. A considerable proportion of reaches (20%) into contralateral space were still executed with the dominant hand. Gabbard and Rabb [8] concluded that hand selection is largely driven by a constant motor dominance or limb preference, and a context-dependent attentional factor.

According to the *kinesthetic hypothesis*, this attentional factor focuses on the proximity of the closest effector to the target and the minimization of a movement’s degrees of freedom to choose among alternative movements [9–13]. The alternative *hemispheric bias hypothesis* states that each hand is more likely to be used in its ipsilateral space because speed and accuracy are greater in ipsilateral space [5, 14–17].

Investigating non-spatial factors contributing to hand selection, Gabbard et al. [18] introduced the concept of task demand. They blindfolded participants and had them pick up boxes and release them according to auditory cues. The locations for pick-up and release were exactly opposite of each other, mirrored at the body midline (e.g. pick up at -70° and release at +70°). In their first condition, the first cue indicated the pick-up location and the second cue indicated the release location. This sequence was reversed in the second condition, i.e. first tone indicated release location. In both conditions, right-handers primarily used their right hands to reach for targets in right space (at a rate of 96%) independent from the sequence of cue presentations.

However, in this experiment dominant hand use in contralateral space stayed relatively high (60–70%), in comparison to the 20–40% that was found in their previous, less demanding visual experiment [7]. Both experiments [7, 18] showed a strong bias of dominant hand use even in contralateral space with an increased use of the dominant hand for reaching to contralateral space in blindfolded participants [18]. The authors concluded: “it appears that when performing a more difficult task requiring deeper processing, the tendency is for subjects to revert to the use of their dominant limb, even when it necessitates reaching contralaterally.” ([18], p. 150).

Przybyla et al. [6] varied the availability of visual feedback to the participants. In contrast to Gabbard et al. [18], their participants were only deprived of visual feedback of their movements with full view of the target positions. Gabbard et al.’s [18] conclusion that an increased use of the dominant arm in contralateral space might be a response to increased task difficulty would let us expect that the elimination of visual feedback–making the task more difficult–should have had a similar effect in Przybyla et al. [6]. Just on the contrary, they observed a reduced dominant hand use in the contralateral hemispace without visual feedback.

Przybyla et al. [6] calculated a reaching frequency midline, indicating the horizontal position at which participants showed an equal proportion of right and left hand use. The reaching frequency midline was always shifted into the hemispace contralateral to the dominant, right hand with a smaller shift in the no-feedback condition. Moreover, Przybyla et al. [6] observed that subjects used the dominant hand significantly more often as targets were located farther away from the starting position in depth. This finding confirmed a very early observation by Baldwin [19], who studied his daughter’s reaching preferences in infancy. He found that the farther away an object was while on the midline, the more likely the dominant hand would be chosen.

Thus, in terms of task demand or effort of reaching, Przybyla et al. [6] reported two opposing outcomes. Reduced dominant hand use and smaller shifts of reaching frequency midline positions in contralateral space was found when visual feedback was eliminated, whereas increased dominant hand use and larger shifts of reaching frequency midline in contralateral space was found with increased reaching distance in depth.

Przybyla et al. [6] resolved this apparent contradiction within the framework of Sainburg’s dynamic dominance hypothesis [20]. Whereas the dominant arm and hand achieve better coordination by predictive control, depending on visual information, the non-dominant system relies more on proprioceptive inputs. This specific change in sensorimotor demands would then modulate the motor dominance factor depending on the availability of visual feedback. The effect of target distance in depth was independent from this sensorimotor factor and–without an explicit interpretation offered by Przybyla et al. [6]–could be interpreted as increased effort, in agreement with Gabbard et al. [18] and Baldwin [19]. An increased preference for the dominant hand due to a rather unspecified increased effort of movement would also agree with findings from Stins et al. [21].

The latter mentioned study analyzed hand selection in a reaching task, comparing reaching for empty glasses with reaching for half-filled glasses in right-handers and left-handers. In agreement with the effort hypothesis, they observed a shift of dominant hand selection towards the contralateral space for half-filled glasses in comparison to empty glasses. However, it remained unclear whether this was due to the increased weight or higher demands on movement coordination with the filled glasses.

Keeping the hand selection results from previous studies in mind, along with the ambiguity of effort, we were determined to update experimental methodology with context to virtual reality (VR) and rehabilitation. To do so, we developed a low-cost, quick, reproducible, and robust hand selection paradigm using VR, and then used this paradigm to measure effort-related effects on hand selection.

Effort of reaching was manipulated through the variation of target distance in height and the application of an additional weight to the non-dominant hand. Based on Przybyla et al.’s [6] observations for the effect of target depth, we expected that increased target height results in a shift of reaching frequency midline positions towards the contralateral space. Based on Stins et al.’s [21] findings, we would expect a similar main effect for an additional weight on the non-dominant hand, in that the dominant hand would be used more often.

Besides this direct investigation of hand selection, we used this paradigm as a test case for our virtual reality paradigm. The successful detection of significant effects would be an important validation that proves a sufficiently realistic situation for the occurrence of ecologically valid effects in hand selection.

In the last two decades, virtual reality (VR) has begun to change the field of neurological rehabilitation and demonstrated its potential in several clinical studies [22–27]. The flexibility of VR-environments, together with the potential to create highly motivating tasks and procedures, makes VR a potentially powerful tool to measure, diagnose, and rehabilitate motor and cognitive impairments [28]. However, clinically certified effective systems often cost over €10,000. We propose a paradigm that uses a state-of-the-art VR hand tracker that costs under €100.

Our participants were faced with a virtual environment, in which a grid-like display of cubes appeared in a random sequence one cube at a time. In each trial they were to ‘contact’ the cube with a freely chosen hand and come back to the starting position. The hands were projected into the virtual environment through an infrared hand tracker.

If all things were equal, we could expect the use of right and left hands to fall equally on each side of the midline, however we predict that even in this virtual environment, the results will follow previous studies and show a preference for the dominant hand into contralateral space. We also expect that the further away a target is from the body’s midline, the more likely the participant will use the ipsilateral hand. Additionally, we predict that increasing the factor of effort will increase the use of the dominant hand in contralateral space.

We will test this effort hypothesis in two ways: first, by dividing the stimuli presentation into three levels of height; and second, in a separate experimental condition, by placing a weighted-glove on the non-dominant hand. The rationale behind adding a weighted-glove in this paradigm was to make a simple connection to what a patient with an impaired arm may experience; i.e. more difficulty in doing tasks with that limb. We wanted to see if the paradigm and setup was sensitive enough to reveal corresponding effects on hand selection.

## Materials and methods

### Ethics statement

The study was approved by the local ethics committee of the University Tübingen and the Medical Faculty Tübingen in accordance with the principles expressed in the Declaration of Helsinki. All participants provided their written informed consent.

### Instrumentation

The experimental setup consisted of the Leap Motion hand tracker (LM; Leap Motion Inc. www.leapmotion.com) mounted to a desk, a Windows laptop computer meeting the minimum specifications of the LM, and a custom stimuli program created in Unity3D and C#. The LM consists of three infrared cameras and two CCD (charge-coupled device) cameras and integrates custom written algorithms (i.e. Orion Beta) for tracking capability. As stated by the manufacturer, the LM tracks hands and fingers at up to 120 frames-per second and provides a 135-degree field of view with roughly 244 cubic centimeters of interactive 3D space (61cm x 61cm x 61cm). Additionally, the maximum tracking is 80cm from the device, and the sensor’s accuracy in fingertip position detection is approximately 0.01mm. The cost of the LM at the time of submission is approximately €79.99.

The laptop computer which ran the LM is an XMG A516 running Windows 10 with a NVIDIA GeForce GTX 1060 graphics card, 16GB of RAM, a solid-state hard drive, and an Intel Core i7-7700HQ CPU @ 2.80GHz. The cost of this computer at the time of submission was approximately €1399. This computer greatly exceeded the *minimum system requirements* of the LM, which are stated as 2GB of RAM and an Intel Core i3/i5/i7 processor. A computer of these specifications can be found for well under €500.

Grip strength was measured using the Takei Physical Fitness Test “Grip-D” made by Takei Scientific Instruments Co., LTD. A pair of gloves, called “Powergloves” (S1 Fig), with pockets to place weighted sandbags were used to impose additional weight to the non-dominant hand.

A custom program was written in Unity3D and C# to display stimuli on the laptop screen (www.unity3d.com). Executables and commented source code are available at https://github.com/EricJamesMcDermott/VR_HandSelection, under the GPL v3 license.

An adapted form of the Edinburgh Handedness Inventory (EHI) [29] was administered to measure the left hand/right hand bias of the participants. At the end of the questionnaire, two additional questions were asked regarding the perceived behavioral change in wearing the weighted-glove in the latter half of the experiment. The full questionnaire and exemplary responses can be found in the supplementary data (S2 File). Prior to analysis, we discarded the question regarding “with which hand do you use a broom (top hand)” given an overwhelming uncertainty expressed by participants regarding this question.

### Participants

The study consisted of 30 participants (mean age 27.2y, SD 8.7y, range 19-56y, 7 females). All participants were healthy, with no reported medical problems or signs thereof. All were compensated with €5 at the end of the experiment. Participants must be “right-handed” as defined by scoring higher than the 4^th^ right decile (>74%) on the laterality index in the 13-item augmented EHI. Three participants were excluded from the data analysis: one participant scored lower than the cutoff for right-handed laterality, one reported that he/she was personally “challenged and motivated to use” the weighted-glove more as if in a gym, and one participant reported explicitly that his job requires extensive ambidextrous hand-use of tools despite a 5^th^ right decile EHI score. After these exclusions we analyzed data from 27 participants (mean age 26.9y, SD 9.1y, range 19-56y, 7 females).

### Experimental paradigm

The stimuli were generated on the computer screen and composed of 3D represented yellow cubes with shading. In total, the experiment consisted of 1 training round, followed by 4 experimental rounds of stimuli presentation. In the training round, 10 stimuli were presented, whereas in the experimental rounds, 48 stimuli were presented. In both cases, the positions of the stimuli were predefined in a script, and then position presentation was randomized upon initiation of the round. In total, with questionnaires and grip measurements, the experiment lasted approximately 15 minutes.

**Fig 1:**
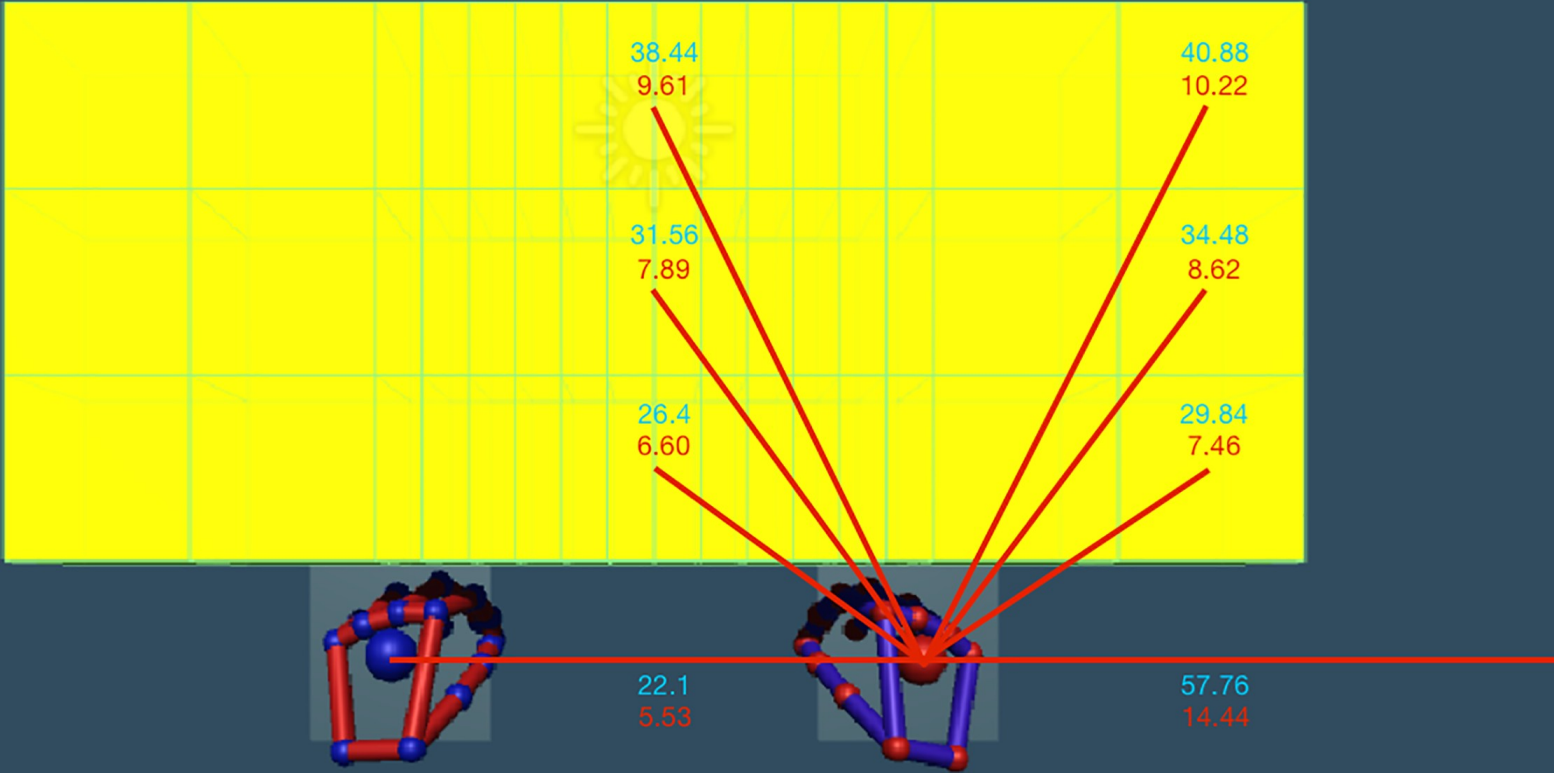
Hand and stimulus configuration. This figure shows all possible stimuli positions on the screen, as well as the initial position of the hands. Each yellow cube is delineated by thin green lines (not present in task) and was presented independently of the others. The red values are virtual 3D space distances on the computer screen in cm, including simulated depth in virtual space. The black values are the distances for corresponding direct movements between respective locations in real 3D space in cm.

To begin, participants filled out personal data and consented to the experimental conditions. After which, hand strength was measured using the Grip-D device starting with the right hand, and then the left hand. Next, participants were instructed to place their elbows on two corresponding and marked points on the table. Now, the training was initiated and the participant could see their virtual hands on the computer screen. Their hands were seen in a ‘bare-bones’ skeletal view, containing a sphere within the palm region. On the screen, two light-blue squares denoted the region where the participant should center the sphere in the middle of their hand. This represented the starting position (Fig 1).

Once the participants were comfortable in this starting position, they were told about the Leap Motion hand tracker in front of them, and that it functioned best when the participants showed it their palms. Once this was made clear, the participants were told: “yellow cubes will be appearing in the environment one at a time, and you are to reach out and contact the cubes freely with whatever hand that you want to, and the object of the task is to do so as fast as possible.” After the participant finished contacting the first stimulus presented in the training round, they were instructed to come back into the starting position and repeat this sequence for every stimulus. The next stimulus was presented exactly 2 seconds after the contact to the previous stimulus to provide enough time to return to the starting position while still keeping attention engaged. After the training run of 10 trials, a break screen appeared, and the participants were asked if they were ready to go onto the next level, and in this case, the experimental round.

In the experimental condition, the stimuli presentation procedure was the same: the participants started from the starting position, reached out with their chosen hand to contact a cube, returned to the starting position, and then the next cube was presented exactly 2 seconds after contacting the previous one. This was repeated until the break screen came up reporting the end of the round. This break screen consisted of a button that the experimenter would click to initiate the next round, as well as a score that consisted of the summation of centimeters per second that the participant moved in contacting the cubes. This score was explained as the summation of the speed of their movements and the participants were told they would be able to see their scores after every round, but no explicit encouragement nor instruction to improve their score was given.

The stimuli positions in each experimental condition were in a 3-row x 13-column grid. Cube presentations in the middle 3 columns were repeated twice per row in each round, each other column was presented once per row, resulting in 48 trials per round. Each stimulus was a 1x1x1 cube in Unity3D units (which translated to a gain-factor of 2.4 on the computer screen; i.e. 2.4cm x 2.4cm x 2.4cm). The distance between the middle of a cube between two adjacent rows was 1 unit, meaning that the top of a cube in row 1 (the lowest row) would perfectly contact the position of the bottom of a cube in row 2. The distance between columns in Unity3D units was as follows: 1–1 - .25 - .25 - .25 - .25 - .25 - .25 - .25 - .25–1–1, meaning that the outermost 2 cubes on the left and right were bordering each other, while the rest had a 75% overlap with their neighboring cube (Fig 1). Exact grid positions in Unity3D units can be found in the supplementary material (S1 File).

Two experimental rounds were conducted consecutively, two more were conducted with the modification that the participant was instructed to wear two gloves, the right glove contained no additional weight, while the left glove contained a 500-gram sandbag. The sandbag was placed in a pocket on top of the glove, which appeared ‘inflated’ with or without the sandbag, resulting in a very similar visual appearance. The weight was distributed equally across the back of the hand (not including the fingers) (S1 Fig). The participant was then instructed again: “you are to reach out and contact the cubes freely with whatever hand that you want to, and the object of the task is to do so as fast as possible.” The participant was asked if they were ready, and then the next round was initiated. Following these two experimental rounds with gloves, this portion of the experiment was concluded, and the participant filled out the Edinburgh Handedness Inventory (EHI) and answered complementary questions on their perception of the weighted-glove.

### Experimental setup

The laptop screen measured 19.4cm by 34.4cm. Two light blue squares (2.3cm x 2.3cm on the computer screen) represented the ‘starting positions’ for XY-space, areas in which the respective hand should be centered within at the start of each trial. Centered in the starting positions, the hands were presented 5.5cm away from each other on the screen, and 14.4cm from the left and right edges of the screen (Fig 1). The actual starting positions of the participants hands were in a different Z-plane in depth from the target stimuli, an average of 5.37cm away from the target stimulus plane due to the slight variations in arm length. Stimulus positions on the screen as well as measured kinematic data from the LM tracker were all in arbitrary Unity3D coordinates and were analyzed as such (please see below ‘Data Collection and Statistical Analysis’). We later converted results from Unity3D coordinates to visual screen (2D and virtual 3D) and real space 3D coordinates to allow for a better understanding, interpretation, and direct comparisons with previous studies.

**Fig 2:**
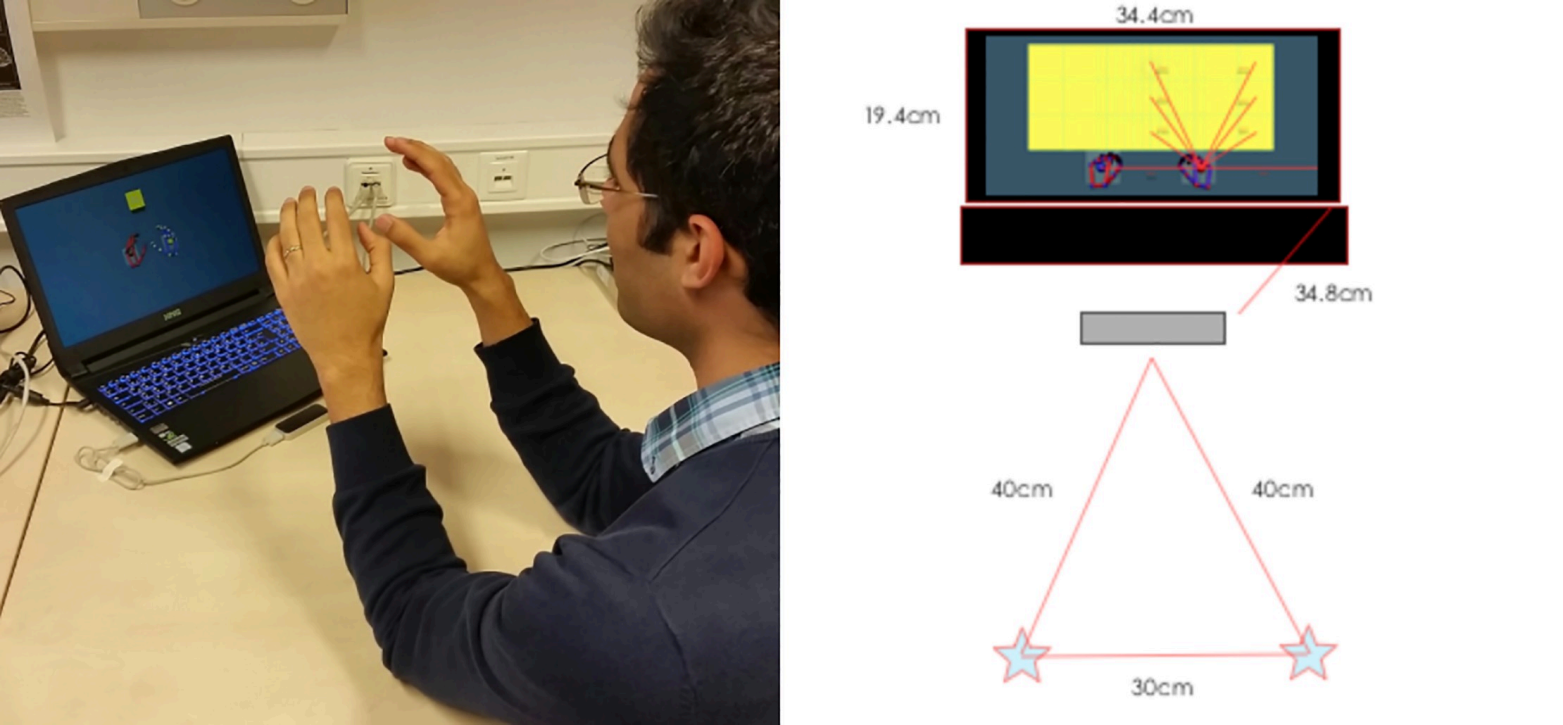
Experimental setup. The left panel shows a participant in the “initial starting position” before going to contact the cube. The right panel shows the same setup in a schematic drawing with the distances on the setup. Respectively, the stars are where the elbows were placed, the grey rectangle is the Leap Motion, and the black rectangles are the keyboard and computer monitor.

In the following, we report distances and positions in the experimental setup in two reference frames, visual 2D and virtual 3D space. First, in correspondence with the 2D visual space of the laptop screen to describe the actually visible stimulus movements on the screen (visual2D). Second, in correspondence with the virtual 3D space considering the simulated visual depth of hand and target representations on the screen (virtual3D). The distance from the hand starting position of either hand to the center of the box in the bottom row 1 of the middle column was 4.2cm in visual2D, and 6.60cm in virtual3D (Figs 1 and 2); reaching distance to the center of the box in the middle row 2 of the middle column was 5.8cm in visual2D, and 7.89cm in virtual3D; reaching distance to the box in the top row 3 of the middle column was 7.9cm in visual2D, and 9.61cm in virtual3D. The distance to reach to the middle of the box in row 1 of the most peripheral column was 4.6cm in visual2D and 7.46cm in virtual3D; reaching distance to the box in row 2 of the most peripheral column was 6.3cm in visual2D and 8.62cm in virtual3D; while the distance for the box in row 3 of the most peripheral column was 8.5cm in visual2D and 10.22cm in virtual3D.

In the third reference frame, real-space 3D (real3D), the hands were held 22.1cm away from each other in correspondence with the starting positions presented on the screen. The elbows were placed at markers on the table exactly 30cm from each other (Fig 2). Each participant was instructed to maintain an arm angle that resulted in a comfortable position, while also extending forward from the elbows and placing the hands slightly inwards so that the center of the virtual palm resided in the starting positions. This position was visually confirmed by the experimenter, who was present at all times. To calculate real3D reaching distances we used a rounded gain factor of 4 for all participants, which was found by dividing the real3D distance the hands were held apart in real space (22.1cm) and the average distance the visual representations of the hands were held apart on the computer screen in virtual3D (5.53cm). Therefore, the calculated real3D distance to the center of the boxes within the middle column of row 1 was 26.4cm; row 2, 31.56cm; and row 3, 38.44cm. (Please see Figs 1 and 2 for further measures.) The LM tracker was positioned 34.8cm diagonally from the bottom corners of the computer screen, and 40cm diagonally from each elbow marker (Fig 2).

### Data collection and statistical analysis

Kinematic data was collected in real-time during the entire duration of the experiment using the LM tracker and custom-written Unity3D scripts. Information that was collected included: 3D coordinates of both hands upon stimulus presentation, timestamp upon stimulus presentation, coordinates of stimulus presentation, 3D trajectory of the hands until contacting a cube at a 20Hz sampling rate, timestamp upon hand contact with stimulus, and which hand contacted the stimulus. Additionally, we recorded EHI scores, grip strength of both hands, and questions regarding the experience wearing the weighted-glove. We chose EHI and grip strength measurements as complementary measurements of handedness because these have been widely used before (for an overview, please see Scharoun and Bryden [30]) and represent two different operationalizations of handedness, independent from our experimental measurements.

Data was used from the experimental rounds only; the training round was not included in the analyses. Within each row, condition, and participant, we sorted stimuli according if they were touched with the left or the right hand. The horizontal distance from midline was averaged across all cubes contacted with either the right or the left hand, resulting in left hand and right hand horizontal mean positions. The sum of these means was first divided by 2, then by the total range of horizontal stimulus positions within the experiment and multiplied by 100. The resulting number estimated the virtual point at which hand-use switched from one hand to the other on a continuous scale, i.e. the reaching frequency midline position (RFM) within each row.

An RFM was determined for each participant at each height, i.e. row of stimuli, and for each condition, i.e. weighted and non-weighted. We then analyzed RFMs with a 3 x 2 factorial repeated-measures ANOVA for the main effects of height (bottom, middle, & top row) and weight (non-weighted vs weighted), and their interaction. Significant findings for the main effect of height or the interaction of weight and height were followed up with subsequent rANOVAs and paired *t*-tests. Bonferroni-corrections for multiple comparison have been applied to the respective post-hoc tests. Assumptions of sphericity have been tested with Mauchly-tests and Greenhouse-Geisser corrections have been applied to DFs in case of a significant violation (Mauchly-tests p < .05).

Please note that all measurements, calculations, and analyses were conducted with Unity3D coordinates. In doing so, we avoided any variability or systematic biases from rounding errors. Only final group results were converted to the three reference spaces, i.e. visual2D, virtual3D, and real3D space.

## Results

**Fig 3:**
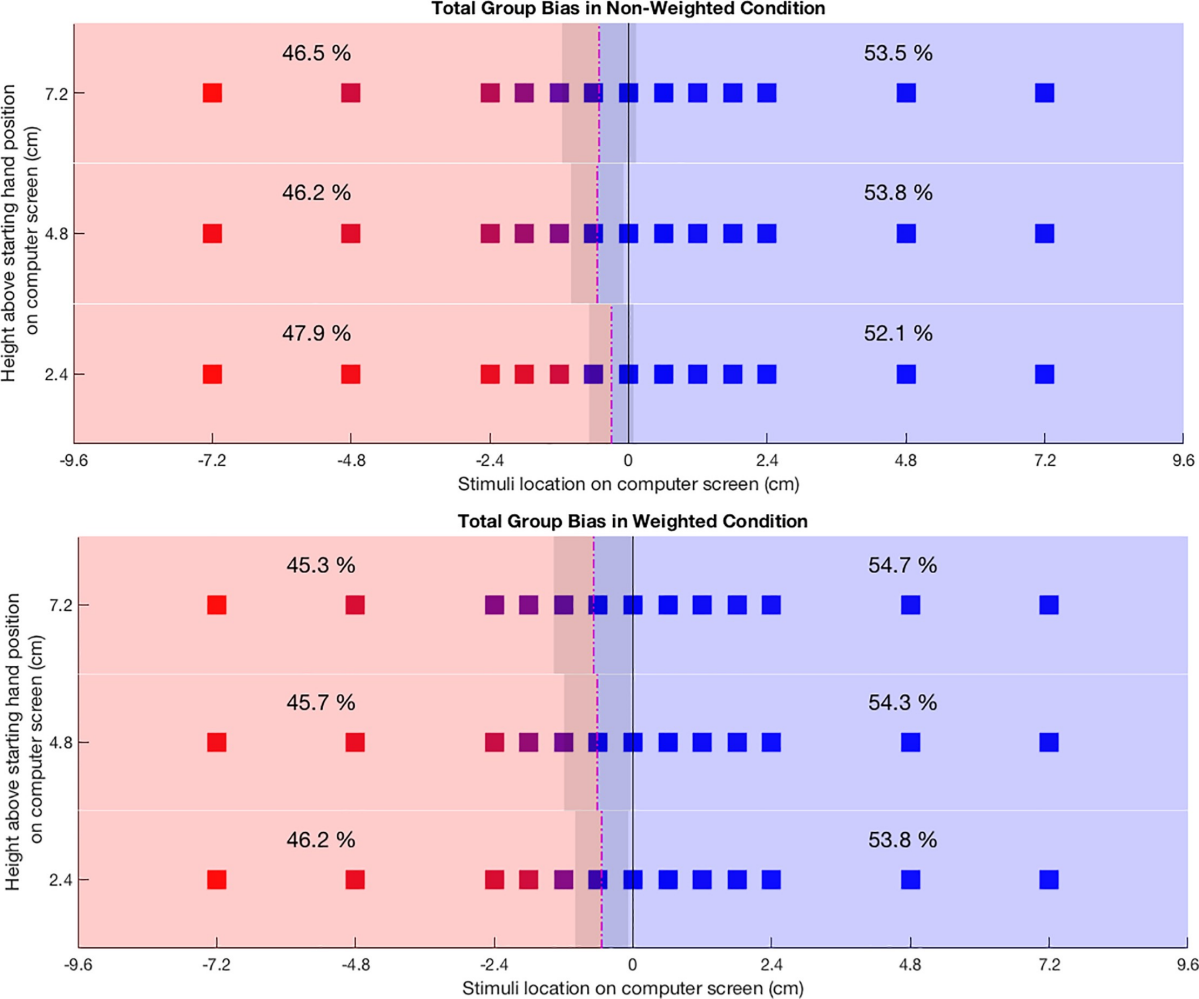
Average reaching frequencies and reaching frequency midline positions. Each cube represents a stimulus location. Group means of hand selection at stimulus positions are color-coded with red squares indicating 100% left hand selection and blue squares indicating 100% right hand selection. Mean percentages of left and right hand use are presented in each hemispace. Dash-dot lines indicate mean reaching frequency midline positions with shaded areas representing their standard deviation. Upper panel: non-weighted conditions; lower panel: condition with weighted left hand glove.

The rANOVA showed a significant main effect for non-weighted (NW) versus weighted (W) conditions (p = 0.009, F_1,26_ = 7.9582; Table 1 and Fig 3) on RFM, with a mean difference (NW—W) of -0.64cm, SEM 0.06 in real3D space; i.e. RFM positions moved significantly towards the left from the non-weighted to the weighted conditions. We also found a significant main effect of height (p = 0.0107, F_2,52_ = 4.960). We followed this up with three paired *t*-tests comparing RFM positions between height levels. These analyses revealed a significant difference between the bottom vs middle row (mean difference bottom—middle: 0.64cm, SEM: 0.064, p = 0.0006) and the bottom vs high row (mean difference bottom—high: 1.04cm, SEM: 0.112, p = 0.0305), with a non-significant result for the comparison of middle vs high (mean difference middle—high: 0.4cm, SEM: 0.104, p = 0.7932). After correcting for multiple comparisons using a Bonferroni-corrected error probability threshold of α = 0.05/3 = 0.0167; only the bottom vs middle row stayed significant. The interaction of the factors height and weight was not significant (p = 0.3528, F_2,52_ = 1.0630).

**Table 1:** Mean reaching frequency midline positions and standard errors of the mean (SEM).

		Non-Weighted	Weighted	Mean (SEM)
**Top row**	**Real3D**	-2.04cm (0.492)	-2.72cm (0.532)	-2.38cm (0.464)
	**Visual2D**	-0.51cm (0.123)	-0.68cm (0.133)	-0.594cm (0.116)
	**Unity3D**	-0.2125 (0.0514)	-0.2838 (0.0553)	-0.2481 (0.0483)
**Middle row**	**Real3D**	-2.16cm (0.348)	-2.48cm (0.444)	-2.32cm (0.360)
	**Visual2D**	-0.54cm (0.087)	-0.62cm (0.111)	-0.58cm (0.090)
	**Unity3D**	-0.2257 (0.0362)	-0.2568 (0.0462)	-0.2413 (0.0376)
**Bottom row**	**Real3D**	-1.20cm (0.296)	-2.16cm (0.352)	-1.68cm (0.296)
	**Visual2D**	-0.30cm (0.074)	-0.54cm (0.088)	-0.42cm (0.074)
	**Unity3D**	-0.1240 (0.0307)	-0.2255 (0.0368)	-0.1748 (0.0309)
**Mean (SEM)**	**Real3D**	-1.80cm (0.340)	-2.44cm (0.4)	-2.04cm (0.352)
	**Visual2D**	-0.45cm (0.085)	-0.61cm (0.1)	-0.51cm (0.088)
	**Unity3D**	-0.1874 (0.0353)	-0.2554 (0.0416)	-0.2214 (0.0367)

**Fig 4:**
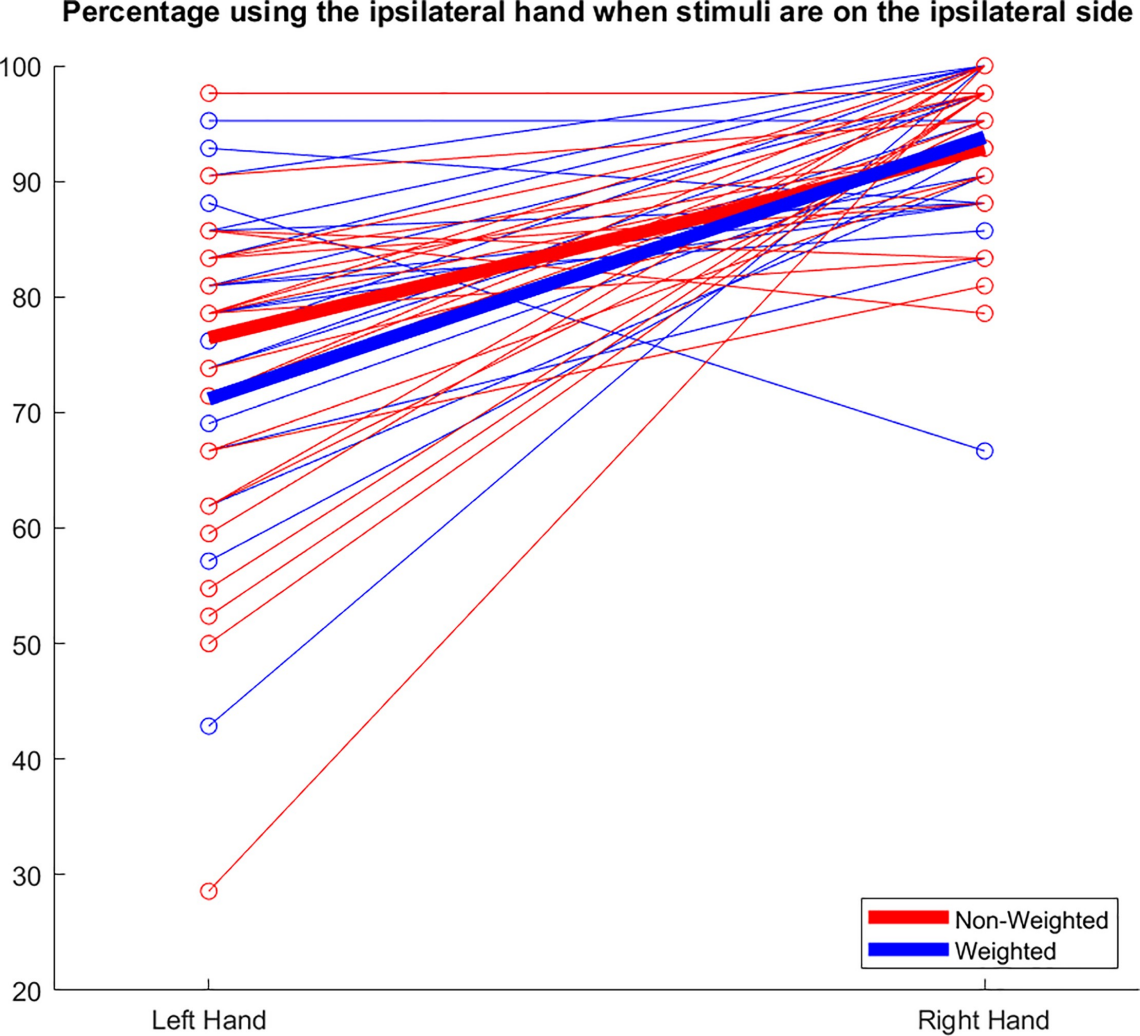
Ipsilateral hand selection. Percentage of selection of left and right hand for their respective ipsilateral targets. The mean values across the group are shown in thick red and blue lines. Individual values from each participant, averaged across trials, are shown as thin, red and blue lines.

We conducted complementary analyses to explore the participants’ behavior and compare our data to previously reported findings and calculated the frequency of ipsilateral hand use in ipsilateral space. This data showed that the participants used the dominant right hand on average 93% of the time in ipsilateral space, whereas the non-dominant left hand was used on average 76% in ipsilateral space (Fig 4). Beyond this difference, variability of ipsilateral hand use across the group was higher for the non-dominant left hand than for the dominant right hand (Fig 4).

**Fig 5:**
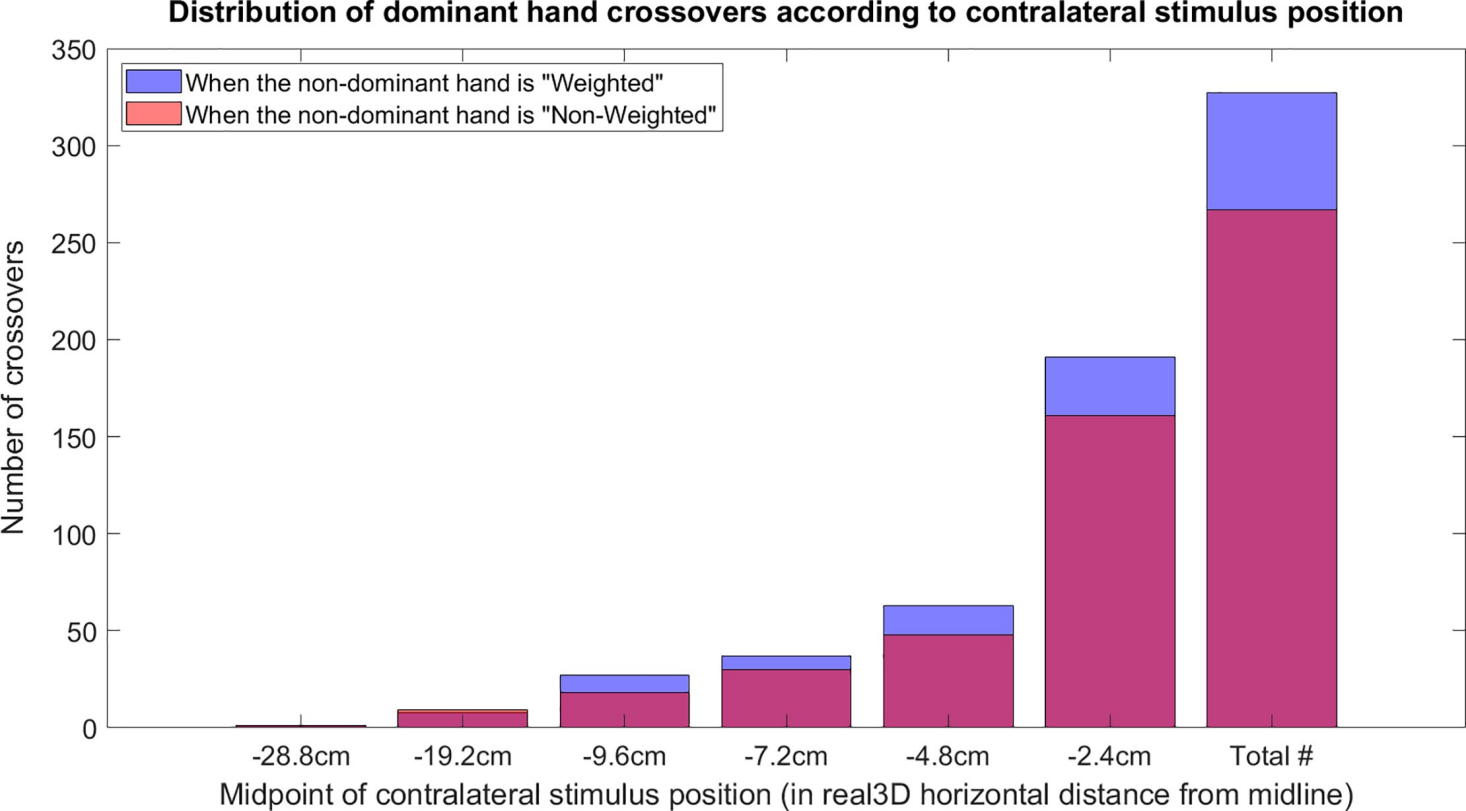
Spatial distributions of dominant (right) hand crossovers. Real 3D space horizontal stimulus positions of left-from-midline, contralateral target stimuli at the three heights positions have been summed up across the group; the red bars represent crossovers in the non-weighted condition, while the blue bars represent the dominant hand crossovers when a weighted-glove was on the non-dominant hand.

We looked at the number of crossovers at each position. In total across the group, there were 267 right-handed crossovers in the non-weighted condition, and this number increased 22.5% to 327 total crossovers in the weighted condition (Fig 5). It also can be seen that there is an exponential decline in crossovers as the targets become farther away into the contralateral space. Comparatively, more crossovers can be seen in the weighted condition until the stimuli become so far out that the number over crossovers in total falls below 10 (at -19.2cm).

**Fig 6:**
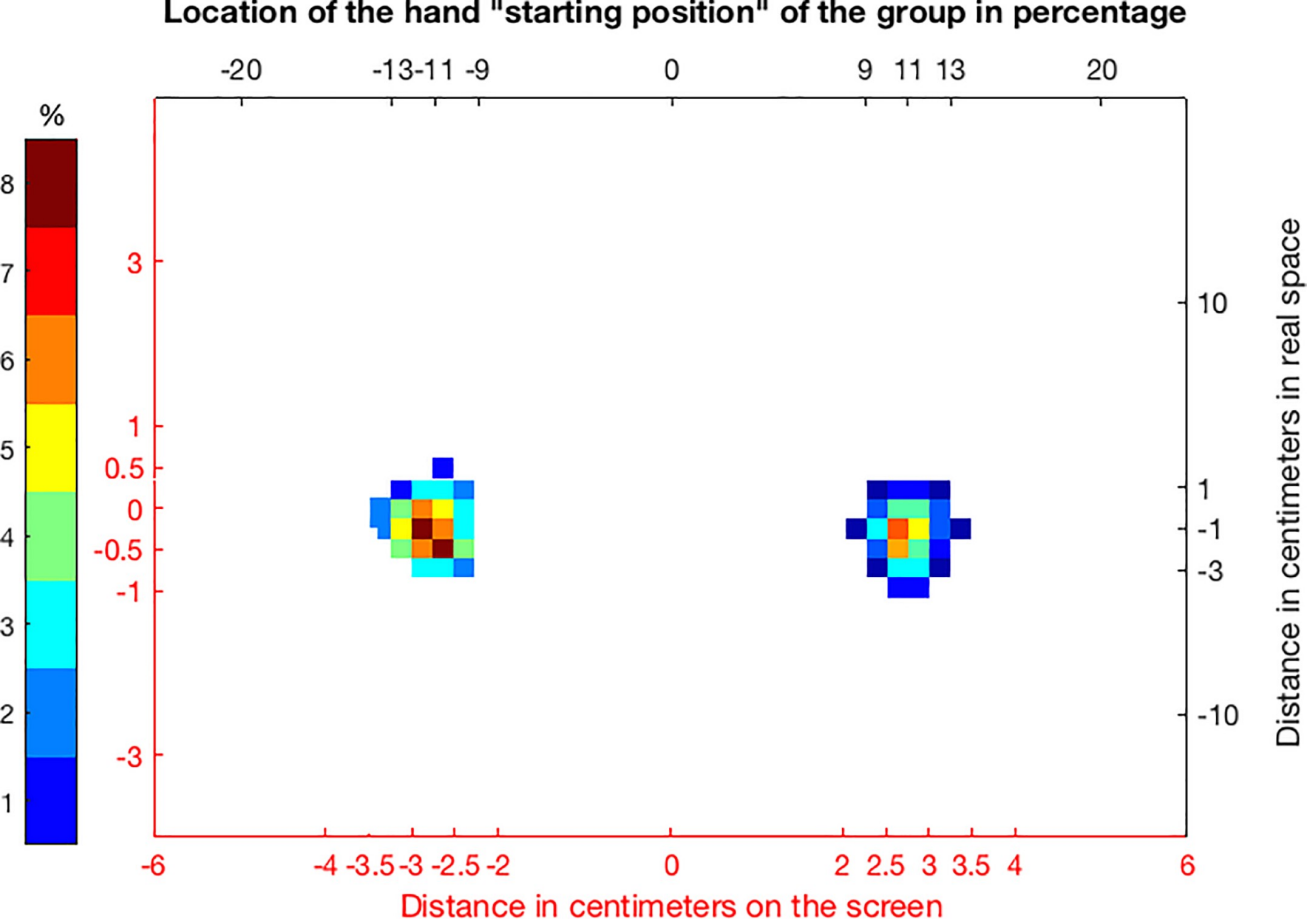
Mean probabilities of hand locations at the start of a trial. The plot shows the mean probability of a given hand location at the start of a trial across participants. Red axes indicate positions in centimeters on the screen (visual2D), while black axes indicate positions in real space (please refer to Figs 1 and 2).

To determine if all participants complied with the instructions and started in the same locations, we analyzed starting positions across the group. For each participant we read out horizontal and vertical coordinates of both hands at the time of target presentation for each trial, resulting in a 2D histogram of probabilities of a hand’s presence at a given location. We averaged these probabilities for each location across the group (Fig 6). This analysis showed that all trials began with the left hand at a starting position between -3.5cm and -2.25cm in the horizontal dimension of visual2D space and -0.8cm and 0.6cm in the vertical dimensions, and the right hand at a starting position between 2cm and 3.5cm (horizontal) and -1cm and 0.4cm (vertical). These data showed that all participants successfully positioned their hands on the instructed starting locations and demonstrated little variation of these starting locations (compare Figs 1 and 2).

The average score on the EHI was 92.02, with a standard deviation of 8.6 and a range of 80.77–100. Average grip strength for the left hand was 39.6 kg (SD 10.7), for the right hand 43.2 kg (SD 10.4) with an average difference between hands (left-right) of -3.6 kg (SD 4.0). We correlated handedness score, RFM measures (each weight condition at each height level and their respective difference values between weighted and non-weighted condition), and grip strength data (grip strength left, grip strength right, difference between grip strengths) across our group of participants. This exploratory correlation analysis comprised 13 variables and thus resulted in 78 correlation coefficients. The Bonferroni-corrected threshold for these analyses was α = 0.05/78 = 0.000641. Only correlations within groups of measurements, i.e. between switch points from different conditions and/or rows, survived this threshold (Table 2). If we also took uncorrected findings into account, we saw two significant correlations between grip strength measures and switch points (grip strength left and switch points weighted condition, high row; grip strength difference and switch point difference between conditions, middle row; Table 2). With a negative sign, both correlations indicate an expected direction of associations, the stronger the left hand (relative to the right hand), the more rightward the position of the (change of the) switch points. Even at uncorrected thresholds we found no correlation of EHI scores, neither with grip strength measures nor with any switch measures. However, this might be because 12/27 participants reached a maximum EHI score of 100 and thus the variation of EHI scores was rather low.

**Table 2:** Significant correlations between dependent variables of hand use.

		gripR	RFM NWM	RFM NWH	RFM WL	RFM WM	RFM WH	RFM DL	RFM DM	RFM DH
**gripL**	**p**	**0.926**					-0.41			
	**r**	4*10^**−12**^					0.0322			
**gripD**	**p**								-0.39	
	**r**								0.0472	
**RFM NWL**	**p**		**0.73**	**0.64**	**0.68**	**0.74**	0.57			
	**r**		1*10^**−5**^	**0.0003**	**0.0001**	1*10^**−5**^	0.0020			
**RFM NWM**	**p**			**0.71**	**0.66**	**0.66**	**0.68**			
	**r**			3*10^**−5**^	**0.0002**	**0.0002**	**0.0001**			
**RFM NWH**	**p**				**0.67**	**0.68**	**0.64**			
	**r**				**0.0001**	9*10^**−5**^	**0.0004**			
**RFM WL**	**p**					**0.83**	**0.63**	-0.58	-0.41	
	**r**					6*10^**−8**^	**0.0004**	0.0016	0.0327	
**RFM WM**	**p**						**0.72**		**-0.63**	
	**r**						2*10^**−5**^		**0.0004**	
**RFM WH**	**p**									-0.49
	**r**									0.0087

Please note that only correlations with p < 0.05 uncorr. are presented.

Significant findings after Bonferroni-correction are shown in bold.

gripL: grip strength left; gripR: grip strength right; gripD: grip strength difference; RFM: reaching frequency midline position; NWL: non-weighted low; NWM: non-weighted middle; NWH: non-weighted high; WL: weighted low; WM: weighted middle; WH: weighted high; DL: difference between weighting conditions low; DM: difference between weighting conditions middle; DH: difference between weighting conditions high.

## Discussion

The main effect of the additional weight applied to the non-dominant hand supported our hypothesis that an increased amount of effort would shift the reaching frequency midline position farther into the contralateral space of the dominant hand. This significant group effect demonstrated the sensitivity of our VR paradigm for the detection of induced changes of hand selection. To allow for comparisons to previous studies on hand selection, we also calculated the frequency of ipsilateral hand use in ipsilateral space. In the non-weighted condition, the participants used the dominant right hand 93% of the time in ipsilateral space, whereas the non-dominant left hand was used 76% in ipsilateral space (Fig 4). Being such, it also follows that this paradigm is representative of the literature in distinguishing hand selection. A study by Harris and Carlson [31] found that the dominant right hand was used 90% of the time with ipsilateral stimuli, where the non-dominant left hand was used 60–75% of the time with ipsilateral stimuli.

Our paradigm implied spatially congruent feedback of hand and arm use in the real world, whereas its translation into the VR world was spatially incongruent with real-world positions and could have resulted in substantial differences in hand selection behavior as reported in previous studies using real-world paradigms. Without a direct comparison between our VR paradigm and an exactly corresponding real-world version of it, we cannot exclude smaller, possibly statistically significant differences. But we can exclude ecologically meaningful, large differences.

We asked our participants if they perceived a change in hand preference from the non-weighted to the weighted condition, 16 of the participants reported that they did notice a change in their behavior. There was a space for follow-up information, short answers were given such as: “It was easier to use the non-weighted hand; it was harder to go quickly with the weighted hand; I used the left hand less”. If a participant gave additional input, it can be found in “S2 File”.

Our main effect of height directly corresponds with the findings by Baldwin [19], who administered over 2000 reach preference trials to his daughter from her 5^th^ month to her 10^th^ month. Baldwin noticed that at a distance of 9in (~23cm), his daughter showed no trace of hand preference. Yet, when he increased the distances to 12-15in (~30-38cm), he recorded a preference ratio of 15:1 for the dominant hand. Baldwin concluded that this selection preference was due to the extra exertion of effort that the farther targets demanded.

Przybyla et al. [6] required participants to reach farther in-depth rather than height, but despite that, their observation was like ours, that the dominant right hand tended to be used increasingly more with increasing depth. Contrary to Przybyla et al. [6], we found no interaction of the two factors distance and weight. This difference might provide new insights into the findings of Przybyla et al. [6] with respect to the dynamic dominance hypothesis.

According to Fitts’ observations [32], movement accuracy decreases with target distance from starting positions. Increasing target distance while keeping movement accuracy constant should then also increase the bias of hand selection towards the hand whose control is optimal under given visual feedback conditions, i.e. right hand with visual feedback, left hand without visual feedback. In contrast, providing full visual feedback throughout our experiment, the participants demonstrated a stable preference for the right hand which was increased by target distance as expected from Fitts’ law [32] combined with Sainsburg’s hypothesis [20].

The absence of an interaction between a change of left hand weight and target distance indicates that simply adding an external weight apparently had no effect on functional asymmetries in neural sensorimotor control [33]. In agreement with observations by Schweighofer et al. [34] the weight added to the left hand in our experiment results in an asymmetric increase of motor effort for the left hand only. With target objects that were large relative to the moving hand and rather loose contact criteria—i.e. the VR target cubes could be touched with any part of the palm to be successful–the motor demands of our paradigm resemble those of Schweighofer et al.’s [34] large target condition.

In summary, the main effect of height observed in our study can be interpreted through a combination of Fitts’ law [32] and Sainsburg’s dynamic dominance hypothesis [20]: the farther away a target of constant size would be, the more accurate a movement needs to be, and under full visual feedback conditions movements with the right hand would be optimal for task success. The main effect of left hand weight can be explained by an asymmetric increase of motor effort for the left hand only.

Weighted and non-weighted blocks were not counterbalanced in our experiment. Non-weighted blocks were always measured first, followed by the weighted blocks. We cannot exclude temporal order effects confounding the presumed effect of weight in our paradigm. To address this critique, we conducted a block-wise analysis of our primary variable, reaching frequency midline position, and found a clear shift from the 2nd non-weighted to the 1st weighted condition. In addition, this analysis showed an absence of any substantial changes within same condition weighted or non-weighted blocks (S2 Fig). This pattern supported our conclusion that primarily the additional weight at the non-dominant hand caused the observed leftward shift of the reaching frequency midline.

Hand preference as such is often measured by questionnaires like the EHI [29], which we also used to select a homogenous group of right-handers. Obviously, hand preference as a person’s trait should influence hand selection in any given task and therefore should also have influenced hand selection in our task. We did not find any association between EHI scores and other behavioral measures, most likely because of our EHI dependent selection of participants and the resulting skewed distribution of EHI score with ceiling effects. Many earlier studies on handedness measured grip strength and investigated its value as a simple measure of hand use lateralization [30]. Therefore, we also adopted this measure for an exhaustive report of the behavioral context and consequences of our new VR paradigm. Because of heterogenous results in the past [30], we did not expect any specific correlation. A full correlation matrix, including all 78 possible combinations or EHI score, grip strength and reaching frequency midline position provides a detailed overview of the inherent association structure between our measures (S3 File). It mostly confirms previous reports of rather volatile associations between different measures of handedness. High correlations (r > 0.7) were only observed for closely related measures (e.g. grip strength left–grip strength right: 0.93, reaching frequency weighted middle–reaching frequency weighted low: 0.83), with all other cross-measures correlations showing only weak, if any correlations. These numbers remind us that different tests quantify different aspects of handedness and hand selection. While hand selection in our task is for sure influenced by handedness, it cannot be fully explained by it.

### Usability for patients and rehabilitation

Importantly, our paradigm showed that we can detect the difference between the weighted and non-weighted conditions even with such a minimal weight. Given we used a 500g weight on the left hand, it would reason that if we increased this weight, we’d see an even greater increase in right hand use. Or on the contrary, if the weight was added to the dominant hand, there should be a certain amount of weight relative to each participant that would create roughly a 50%/50% switch-point.

The rationale behind adding the weighted-glove in this paradigm was to make a simple connection to what a patient with an impaired arm may experience; i.e. more difficulty in doing tasks with that limb. We wanted to see if the paradigm was sensitive enough to distinguish the within-subject condition of the added weight. Our findings support that the paradigm indeed is sensitive enough to do so.

We used a computer screen for visual presentation, instead of a fully immersive virtual reality setup including VR goggles. Critical features for us were low costs, usability, absence of virtual reality sickness, and avoidance of substantial learning periods before effective use of the system. Commercially available VR goggle systems still do not fully meet all these requirements. It should be noted that in immersive VR, the experience of depth would have been more realistic. However, to make the virtual world an exact copy of the real situation, all spatial dimensions would have to be calibrated for each user. This would add another step before the actual use of the system. Our data show that a simpler and more economical setup suffices for the induction and detection of significant effects even in healthy young participants.

### Future outlook

Neuropsychologists have a vested interest in understanding hand selection and its relation as a direct link into cerebral lateralization and functioning. Given the validation of our paradigm with the previous literature we believe it can be easily adapted in the future to create a reliable diagnostic instrument and rehabilitative training for patients. For example, after gathering the baseline data of a patient and measuring their *switch-point* for each height level, one would have insight into the extent of the reaching ability compared to controls. If we would see a detriment in left hand use, such as the case in many hemiparesis patients, we could implement a rehabilitative training paradigm. In this next-step adaptation, instead of yellow cubes being presented, we could present ‘red’ or ‘blue’ cubes, corresponding to a ‘red-colored’ left, or a ‘blue-colored’ right hand. Through the rules set by the programming of the environment, the paradigm would only recognize when the red cubes would be contacted by the ‘red hand’, and vice versa. Given our complete control over the stimulus presentation, we could, for example, present these cubes at the same locations as the participant contacted in the baseline paradigm. Yet, we can also challenge the patient to utilize the significantly affected left hand by programming a training goal to shift the *switch-point* by a certain percentage. Dependent upon the behavior of the patient (i.e., they were successful), the following trials would shift the *switch-point* farther, challenging the patient once again. This sort of adaptive learning paradigm has been shown to be extremely beneficial in rehabilitative efforts [28].

Furthermore, rehabilitating left hand use is only one of the many adaptations of the basic paradigm structure we created; easy manipulations can encourage patients to increase accuracy, or speed, or contralateral hand use, or attention and visually guided movements on pre-set trajectories. The virtual environment created here offers a viable approach for flexible rehabilitation paradigms, while its combination with real-time adaptive learning algorithms could be used to tailor therapy for each individual patient.

## Supporting information

S1 Fig Visualization of “Powergloves”. The top image shows the glove on the left filled with two sandbags, while the glove on the right has no sandbags inside (displayed outside). The remaining images show a participant wearing the gloves.
(TIF)

S2 Fig Block-wise reaching frequency midline positions. Mean reaching frequency midline positions in Unity3D measures averaged across height levels for each block in sequential order with standard errors of the mean. NW1: 1^st^ non-weighted block, 1^st^ experimental block; NW2: 2^nd^ non-weighted block, 2^nd^ experimental block; W1: 1^st^ weighted block, 3^rd^ experimental block; W2: 2^nd^ weighted block, 4^th^ experimental block.
(TIF)

S1 File List of grid positions of cube presentations in Unity3D units. Note: the X-axis values are centered on 4.
(DOCX)

S2 File Questionnaire and responses. (DOCX)

S3 File Correlation matrix. (DOCX)
