# Supplementary figures and images for: Effects of arm weight and target height on hand selection: A low-cost virtual reality paradigm
Source: PLoS One. 2019 Jun 21;14(6):e0207326. doi: 10.1371/journal.pone.0207326 (PMC6588216; doi:10.1371/journal.pone.0207326)

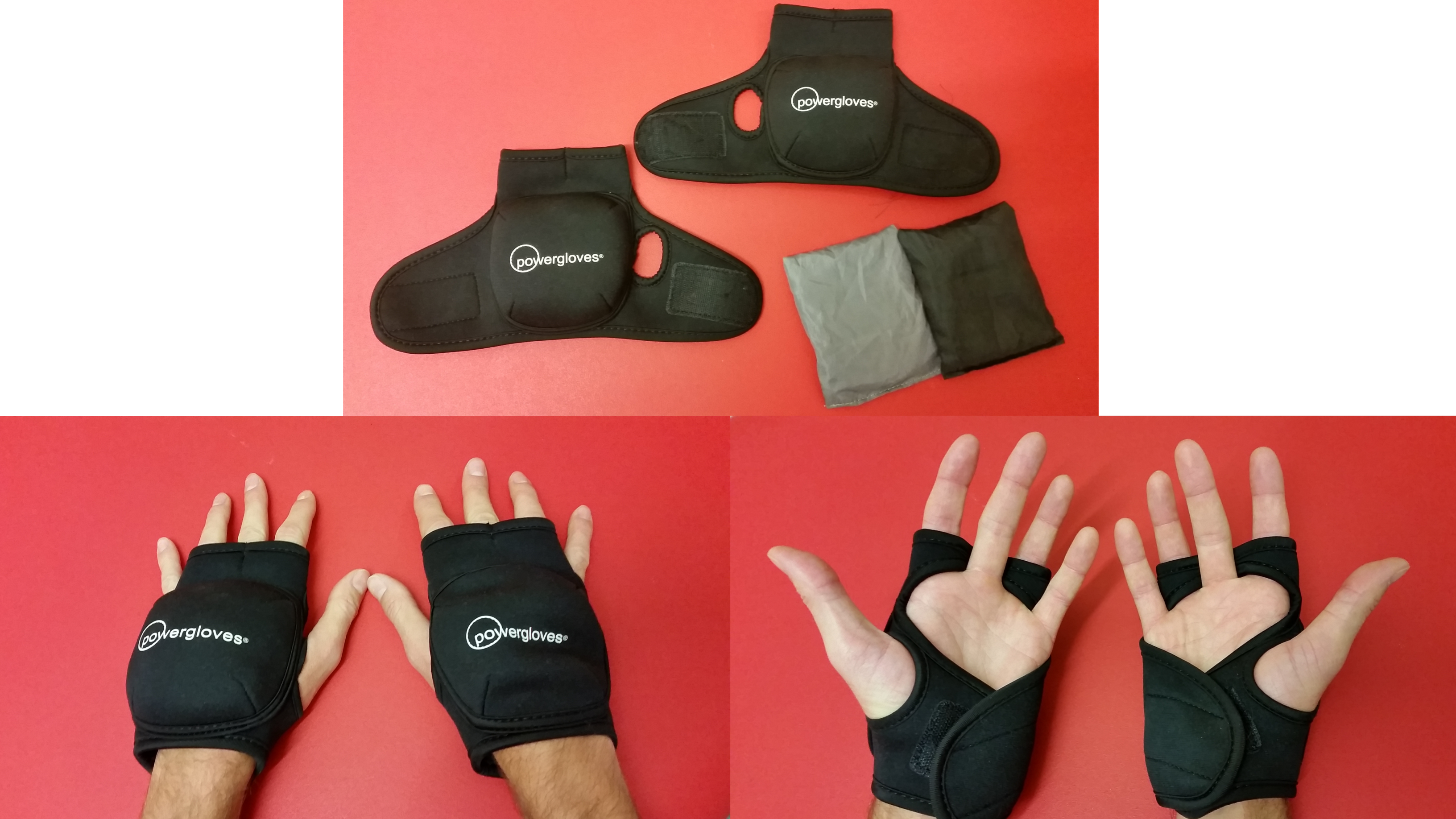

Supplement: S1 Fig — The top image shows the glove on the left filled with two sandbags, while the glove on the right has no sandbags inside (displayed outside). The remaining images show a participant wearing the gloves. (TIF) [file pone.0207326.s001.tif]

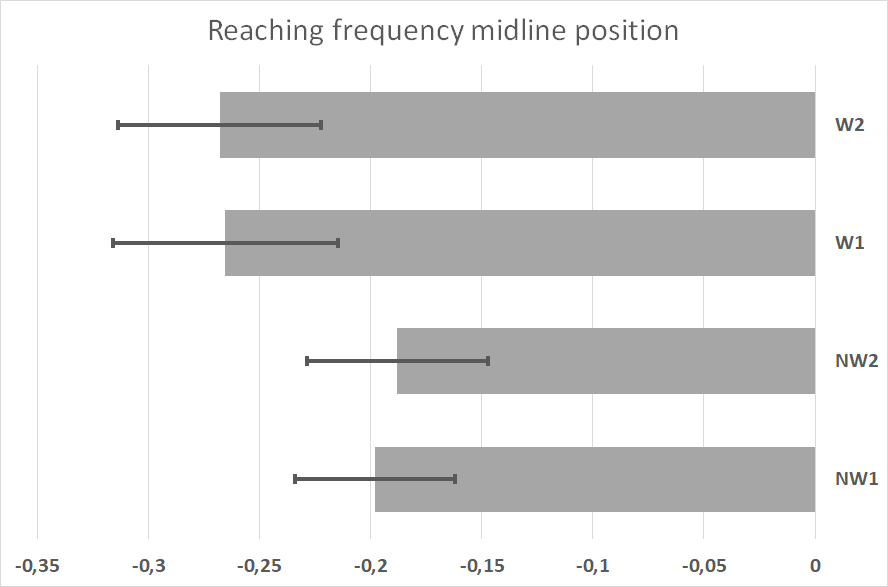

Supplement: S2 Fig — Mean reaching frequency midline positions in Unity3D measures averaged across height levels for each block in sequential order with standard errors of the mean. NW1: 1st non-weighted block, 1st experimental block; NW2: 2nd non-weighted block, 2nd experimental block; W1: 1st weighted block, 3rd experimental block; W2: 2nd weighted block, 4th experimental block. (TIF) [file pone.0207326.s002.tif]
