# Supplementary material for: Effects of arm weight and target height on hand selection: A low-cost virtual reality paradigm
Source: PLoS One. 2019 Jun 21;14(6):e0207326. doi: 10.1371/journal.pone.0207326 (PMC6588216; doi:10.1371/journal.pone.0207326)
Supplement: S1 File — Note: the X-axis values are centered on 4. (DOCX) [file pone.0207326.s003.docx]

**S1 File. List of grid positions of cube presentations in Unity3D units. Horizontal center position is X = 4. All stimuli were presented in the same depth plane (Z = 1).**

| **X** | **Y** | **Z** | **X** | **Y** | **Z** |
| --- | --- | --- | --- | --- | --- |
| 1 | 1 | 1 | 4.25 | 1 | 1 |
| 1 | 2 | 1 | 4.25 | 2 | 1 |
| 1 | 3 | 1 | 4.25 | 3 | 1 |
| 2 | 1 | 1 | 4.5 | 1 | 1 |
| 2 | 2 | 1 | 4.5 | 2 | 1 |
| 2 | 3 | 1 | 4.5 | 3 | 1 |
| 3 | 1 | 1 | 4.75 | 1 | 1 |
| 3 | 2 | 1 | 4.75 | 2 | 1 |
| 3 | 3 | 1 | 4.75 | 3 | 1 |
| 3.25 | 1 | 1 | 5 | 1 | 1 |
| 3.25 | 2 | 1 | 5 | 2 | 1 |
| 3.25 | 3 | 1 | 5 | 3 | 1 |
| 3.5 | 1 | 1 | 6 | 1 | 1 |
| 3.5 | 2 | 1 | 6 | 2 | 1 |
| 3.5 | 3 | 1 | 6 | 3 | 1 |
| 3.75 | 1 | 1 | 7 | 1 | 1 |
| 3.75 | 2 | 1 | 7 | 2 | 1 |
| 3.75 | 3 | 1 | 7 | 3 | 1 |
| 4 | 1 | 1 |  |  |  |
| 4 | 2 | 1 |  |  |  |
| 4 | 3 | 1 |  |  |  |
