# Supplementary material for: Effects of arm weight and target height on hand selection: A low-cost virtual reality paradigm
Source: PLoS One. 2019 Jun 21;14(6):e0207326. doi: 10.1371/journal.pone.0207326 (PMC6588216; doi:10.1371/journal.pone.0207326)
Supplement: S2 File — (DOCX) [file pone.0207326.s004.docx]

The handedness questionnaire has been modified from the original EHI [39], i.e. items 10-13 have been added (<http://www.brainmapping.org/shared/Edinburgh.php>). The answer options for the following EHI questions were “right”, “left”, “no preference”, and “sometimes I use the other hand” (when having selected also “right” or “left”).

1) Writing

2) Drawing

3) Throwing

4) Using scissors

5) Using a toothbrush

6) Using a knife (without a fork)

7) Using a spoon

8) Striking a match

9) Opening the lid of a box

10) Holding a computer mouse

11) Opening a door with a key

12) Holding a hammer

13) Holding a hair comb

Follow-up questions on the weighted gloves with exemplary responses:

1) *While wearing the weighted glove, did you notice a change in your hand preference?*

*If yes, how did it change?*

2) *Did the weighted glove cause any other effect on your behavior?*

| • If the squares came in a central position nearer to my left hand, I tended to use my right hand  • It was easier to just use my right hand  • I changed the hand preference while touching the cube by using my left hand less, sometimes, but not every time  • Didn't want to use the weighted glove because it is a little hard to go as fast as you can, that's why I tried to use the right hand as often as possible, it's easier.  • I used the left one less because of the weight on it  • I used the right hand more because the left one got heavier  • Die beschwerte Hand wollte ich weniger nutzen (wanted to use the weighted-hand less)  • Used the right hand even more  • Noch weniger die Linke als zuvor ([Used] the left hand less than before)  • Towards the end I would switch to the lighter hand  • I think I used my left hand less  • Slower, thought that I preferred the right hand (not sure if true)  • Wanted to use left hand less  • I think I was slower, but before I started I promised myself to use the glove that was heavier even if it was harder but, while I was playing, I simply react spontaneously so I don´t think these influenced the hand preference. The goal was to go fast, and to go fast you have to act spontaneously.  • I was simply thinking which hand was closer to the target, but sometimes I made crossovers  • I noticed that I was using the weighted left less, but not because of a choice.  • Sometimes I thought twice about what hand to use  • Made my left hand tired, maybe less preference to use.  • Noticed the weight on the hand after a little bit --- after many uses of the hand with the weight it was slightly uncomfortable, but not a pain  • Yes, I did move slower with the left hand  • It was more of an exercise for the left hand, it was more difficult.  • Because the goal was to be fast as possible, I tried to avoid the left hand with the weight as much as possible  • Wanted to continue using the hand where I normally would despite knowing it would take more effort than switching hands |
| --- |
