# Supplementary material for: Effects of arm weight and target height on hand selection: A low-cost virtual reality paradigm
Source: PLoS One. 2019 Jun 21;14(6):e0207326. doi: 10.1371/journal.pone.0207326 (PMC6588216; doi:10.1371/journal.pone.0207326)
Supplement: S3 File — (DOCX) [file pone.0207326.s005.docx]

**Supplementary Table 1. All correlation coefficients between dependent variables of hand use.**

|  | **EHI** | **gripL** | **gripR** | **gripD** | **RFM NWL** | **RFM NWM** | **RFM NWH** | **RFM WL** | **RFM WM** | **RFM WH** | **RFM DL** | **RFM DM** | **RFM DH** |
| --- | --- | --- | --- | --- | --- | --- | --- | --- | --- | --- | --- | --- | --- |
| **EHI** |  | 0.20 | 0.18 | 0.07 | -0.11 | -0.29 | -0.26 | -0.21 | -0.28 | -0.36 | 0.16 | 0.06 | 0.14 |
| **gripL** |  |  | 0.93 | 0.27 | -0.01 | -0.17 | -0.21 | -0.20 | -0.01 | -0.41 | 0.25 | -0.16 | 0.27 |
| **gripR** |  |  |  | -0.12 | 0.02 | -0.03 | -0.13 | -0.11 | -0.01 | -0.37 | 0.17 | -0.02 | 0.30 |
| **gripD** |  |  |  |  | -0.10 | -0.37 | -0.22 | -0.25 | 0.01 | -0.15 | 0.23 | -0.39 | -0.07 |
| **RFM NWL** |  |  |  |  |  | 0.73 | 0.64 | 0.68 | 0.74 | 0.57 | 0.21 | -0.21 | 0.03 |
| **RFM NWM** |  |  |  |  |  |  | 0.71 | 0.66 | 0.66 | 0.68 | -0.07 | 0.17 | -0.02 |
| **RFM NWH** |  |  |  |  |  |  |  | 0.67 | 0.68 | 0.64 | -0.18 | -0.16 | 0.36 |
| **RFM WL** |  |  |  |  |  |  |  |  | 0.83 | 0.63 | -0.58 | -0.41 | -0.01 |
| **RFM WM** |  |  |  |  |  |  |  |  |  | 0.72 | -0.29 | -0.63 | -0.11 |
| **RFM WH** |  |  |  |  |  |  |  |  |  |  | -0.20 | -0.25 | -0.49 |
| **RFM DL** |  |  |  |  |  |  |  |  |  |  |  | 0.31 | 0.04 |
| **RFM DM** |  |  |  |  |  |  |  |  |  |  |  |  | 0.13 |

EHI: Edinburgh Handedness Inventory; gripL: grip strength left; gripR: grip strength right; gripD: grip strength difference; RFM: reaching frequency midline position; NWL: non-weighted low; NWM: non-weighted middle; NWH: non-weighted high; WL: weighted low; WM: weighted middle; WH: weighted high; DL: difference between weighting conditions low; DM: difference between weighting conditions middle; DH: difference between weighting conditions high.
